# Systematic review with network meta-analysis: dual therapy for high-risk bleeding peptic ulcers

**DOI:** 10.1186/s12876-017-0610-0

**Published:** 2017-04-19

**Authors:** Keda Shi, Zeren Shen, Guiqi Zhu, Fansheng Meng, Mengli Gu, Feng Ji

**Affiliations:** 1 Department of Gastroenterology, The First Affiliated Hospital, School of Medicine, Zhejiang University, No. 79 Qingchun Rd, Hangzhou, 310000 Zhejiang China; 2 Eye Center, The Second Affiliated Hospital, School of Medicine, Zhejiang University, Hangzhou, China; 3 Department of Hepatology, Liver Research Center, The First Affiliated Hospital, Wenzhou Medical University, Wenzhou, China

**Keywords:** Ulcer bleeding, Endoscopic hemostasis, Mechanical therapy, Thermal therapy, Injection therapy

## Abstract

**Background:**

Adding a second endoscopic therapy to epinephrine injection might improve hemostatic efficacy in patients with high-risk bleeding ulcers but the optimum modality remains unknown. We aimed to estimate the comparative efficacy of different dual endoscopic therapies for the management of bleeding peptic ulcers through random-effects Bayesian network meta-analysis.

**Methods:**

Different databases were searched for controlled trials comparing dual therapy versus epinephrine monotherapy or epinephrine combined with another second modality until September, 30 2016. We estimated the ORs for rebleeding, surgery and mortality among different treatments. Adverse events were also evaluated.

**Results:**

Seventeen eligible articles were included in the network meta-analysis. The addition of mechanical therapy (OR 0.19, 95% CrI 0.07–0.52 and OR 0.10, 95% CrI 0.01–0.50, respectively) after epinephrine injection significantly reduced the probability of rebleeding and surgery. Similarly, patients who received epinephrine plus thermal therapy showed a significantly decreased rebleeding rate (OR 0.30, 95% CrI 0.10–0.91), as well as a non-significant reduction in surgery (OR 0.47, 95% CrI 0.16–1.20). Although differing, epinephrine plus mechanical therapy did not provide a significant reduction in rebleeding (OR 0.62, 95% CrI 0.19–2.22) and surgery (OR 0.21, 95% CrI 0.03–1.73) compared to epinephrine plus thermal therapy. Sclerosant failed to confer further benefits and was ranked highest among the 5 treatments in relation to adverse events.

**Conclusions:**

Mechanical therapy was the most appropriate modality to add to epinephrine injection. Epinephrine plus thermal coagulation was effective for controlling high risk bleeding ulcers. There was no further benefit with sclerosants with regard to rebleeding or surgery, and sclerosants were also associated with more adverse events than any other modality.

**Electronic supplementary material:**

The online version of this article (doi:10.1186/s12876-017-0610-0) contains supplementary material, which is available to authorized users.

## Background

Non-variceal gastrointestinal bleeding is a major cause of hospitalization. Some reports demonstrated a temporal decrease in the incidence of peptic ulcer bleeding, which was thought to be associated with the high eradication rate of *Helicobacter pylori* (Hp) and the widespread use of anti-secretory drugs [1–3]. However, this change was not been seen in all the studies [4, 5]. Moreover, the in-hospital case fatality associated with upper GI complication events has remained constant [3]. Thus, peptic ulcer bleeding remains an important cause of hospital admissions and death. Endoscopic hemostatic therapy has been recommended as a first-line therapy for high risk bleeding ulcers [6].

The currently available endoscopic hemostatic methods include injection therapy, thermal coagulation, and mechanical therapy. Among these approaches, injection of epinephrine is the most popular endoscopic method used to stop bleeding because of its safety, low cost, and easy application. Clinical trials and traditional meta-analyses have showed that adding a second endoscopic therapy to epinephrine injection might improve hemostatic efficacy and patient outcomes [7–9]. Therefore, recent practice guidelines recommend that epinephrine injection not be used as a monotherapy. If used, it should be combined with a second endoscopic hemostasis modality (i.e., an injection agent other than epinephrine, mechanical therapy, or thermal coagulation) [10].

However, due to a lack of head-to-head trials comparing different additional treatments after epinephrine injection, the optimum modality in addition to epinephrine remains unclear [10, 11]. Theoretically, a large-scale clinical trial with multiple comparator arms might address this question. Nevertheless, it is not feasible for any single trial to compare all available treatment options. To establish the optimum epinephrine injection-based dual therapy for high risk bleeding ulcers, we therefore performed a random-effects network meta-analysis to compare the efficacy of major treatment modalities (i.e., epinephrine injection plus sclerosant injection, thrombin injection, mechanical therapy or thermal coagulation) in terms of rebleeding, need for surgery and mortality and also evaluated the complications of these treatments.

## Methods

### Search strategy

This systematic review was reported according to PRISMA (Preferred Reporting Items for Systematic Reviews and Meta-Analyses) guideline [12]. A systematic search of PubMed and Cochrane library databases was conducted using the MeSH search terms “gastrointestinal hemorrhag, and hemostasis, endoscopic” until the end of September 2016. Search strategy was provided (Additional file 1: Search strategy). A manual search was also performed of the bibliographies of the identified publications, including relevant meta-analyses and systematic reviews.

### Selection criteria

Randomized controlled trials were included that met the following criteria: (a) Patients with high-risk bleeding peptic ulcers. High-risk lesions were defined as peptic ulcers with an active bleeding or a non-bleeding visible vessel. (b) Dual therapy (i.e., epinephrine injection plus sclerosant injection, thrombin injection, mechanical therapy or thermal coagulation) compared to epinephrine injection alone or epinephrine combined with a second modality (i.e., injection, mechanical, or thermal therapy). Mechanical therapy included hemoclips and band ligation. (c) One or more of the following outcomes were assessed: rebleeding, need for surgery, mortality, and complications. Eligible studies had to be published as full-length articles written in English.

### Choice of outcomes

Rebleeding (clinical or endoscopic evidence of rebleeding after the first endoscopic treatment) was chosen as the primary outcome of this study because it most accurately reflects the efficacy of endoscopic hemostasis [9, 13]. Secondary outcomes included the proportion of patients who needed a surgery, all causes of mortality (30-day mortality or in-hospital mortality) and complications (i.e., induction of massive bleeding, perforations, and tissue necrosis).

### Data extraction

Two investigators (KS and ZS) independently reviewed the full manuscripts of the eligible studies and extracted information into an electronic database, including the publication data (i.e., the first author’s name, year of publication, and country in which the studies were conducted), the study design (number of patients assigned to each group, interventions, comparisons) and the number of patients with/without outcomes (rebleeding, need for surgery, mortality, complications) in each group. Disagreements were resolved by consensus.

### Study quality

The quality of the methodology was independently assessed by 2 reviewers (KS and GZ) using the Cochrane Risk of Bias Tool, which is an established tool based on assessing the sequence generation for the randomization of subjects, concealment of treatment allocation, blinding, incomplete outcome data, selective outcome reporting and other sources of bias [14]. Disagreements were resolved by consensus.

### Data analysis

Network meta-analysis was performed with a random-effects model within a Bayesian framework using the Markov chain Monte Carlo methods provided by the Aggregate Data Drug Information System (ADDIS) software (http://www.drugis.org/index) [15]. Node split analyses were used to verify the consistency between the direct and indirect evidence [16]. A P-value less than 0.05 for the comparison between direct and indirect effects in the node splitting analysis indicated there was significant inconsistency. If there was no significant inconsistency, a consistency model was used to analyze the relative effects of the interventions. Odds ratios were estimated and reported along with their corresponding CrI. We also assessed the probability that each treatment was the most efficacious modality, the second best, the third best and so on using the ADDIS software, which may be helpful in clinical practice. Therefore, the multiple-treatments meta-analysis increased statistical power by incorporating evidence from both direct and indirect comparisons between all combined therapies. We calculated the percentage contribution of each estimate to the entire network and the results were summarized in the contribution plot as well as the study limitation graph [17, 18]. To assess whether the hemostatic effect of dual therapy were affected by differences in confounding factors between the trials, we performed meta-regression by including major confounding factors, such as the medical treatments used in each study, the routine use of the second endoscopy and the year of publication.

## Results

### Study characteristics

**Fig. 1:**
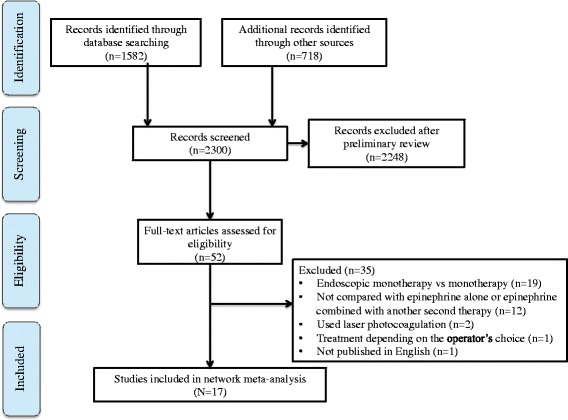
Study selection

We identified 2300 studies in the primary search (Fig. 1). After scanning the title and abstract, a total of 2248 studies were excluded. Of the remaining 52 studies, 35 were further excluded for various reasons after a detailed assessment of the full text (endoscopic monotherapy vs monotherapy (*n* = 19). The reasons included dual therapy was not compared to epinephrine monotherapy or epinephrine was combined with a second modality (*n* = 12), laser photocoagulation was used as thermal therapy (*n* = 2), the treatment depended on the operator’s choice, or the article was not in English. Finally, 17 randomized trials involving 1939 patients who received 1 of the 5 treatment strategies met the inclusion criteria. Table 1 summarizes the characteristics of the studies included in the meta-analysis.

**Table 1:** Characteristics of included studies

Author (Year)	Country	Comparison	No. of Patients	Rebleeding (%)	Surgery (%)	Mortality (%)	Complication (%)
			Treatment/Control	Treatment/Control	Treatment/Control	Treatment/Control	Treatment/Control
Balanzo (1990) [21]	Spain	Epi + Thromb vs Epi	32/32	6/13	16/13	0/0	NR/NR
Pescatore (2002) [20]	Switzerland	Epi + Thromb vs Epi	65/70	22/24	6/10	3/3	2/1
Lin (1999) [33]	Taiwan, China	Epi + Therm vs Epi	32/32	6/34	3/16	3/9	0/0
Park (2004) [9]	Korea	Epi + Mech vs Epi	45/45	4/20	2/4	0/2	NR/NR
Kubba (1996) [19]	Scotland	Epi + Thromb vs Epi	70/70	4/20	4/7	0/10	0/0
Chung (1996) [26]	HongKong, China	Epi + Scler vs Epi	79/81	8/11	11/15	9/5	NR/NR
Lin (1993) [24]	Taiwan, China	Epi + Scler vs Epi	32/32	16/34	6/3	6/0	0/0
Chung (1993) [25]	HongKong, China	Epi + Scler vs Epi	98/98	11/9	14/16	4/9	1/0
Lo (2006) [13]	Taiwan, China	Epi + Mech vs Epi	52/53	4/21	0/9	2/0	0/0
Chung (1999) [22]	Korea	Epi + Mech vs Epi	42/41	10/15	2/15	2/2	0/7
Rutgeerts (1989) [30]	Belgium	Epi + Scler vs Epi	40/40	18/40	8/15	5/10	4/0
Villanueva (1993) [27]	Spain	Epi + Scler vs Epi	33/30	21/10	15/13	3/6	3/0
Sollano (1991) [28]	Philippines	Epi + Scler vs Epi	29/32	7/6	0/3	0/3	3/0
Garrido (2002) [31]	Spain	Epi + Scler vs Epi	40/45	8/27	NR/NR	NR/NR	NR/NR
Choudari (1994) [29]	England	Epi + Scler vs Epi	52/55	13/15	8/7	0/2	NR/NR
Taghavi (2009) [23]	Iran	Epi + Therm vs Epi + Mech	89/83	11/5	2/0	2/1	0/0
Chung (1997) [32]	HongKong, China	Epi + Therm vs Epi	136/134	4/9	6/10	6/5	1/0

*NR* not reported; *Epi* epinephrine injection, *Mech* mechanical hemostasis, *Therm* thermal coagulation, *Thromb* thrombin injection, *Scler* sclerosant injection

**Fig. 2:**
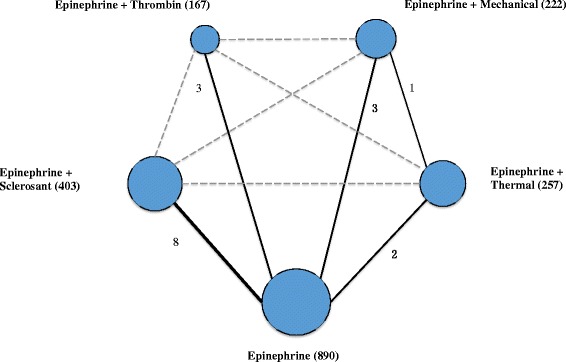
Network of the comparisons for the Bayesian network meta-analysis. Lines connect the interventions that were studied in head-to-head (*direct*) comparisons in the eligible controlled trials, while the interrupted lines connect indirect comparisons. The size of the nodes is proportional to the number of patients (*in parentheses*) to receive the treatment. The width of the lines was proportional to the number of trials (*beside the line*) comparing the connected treatments

Patients were treated with epinephrine plus thrombin in 3 studies [19–21], epinephrine plus mechanical therapy in 4 studies (including three studies used hemoclips alone [13, 22, 23], while the other study used hemoclips as well as band ligation [9]), epinephrine plus sclerosant in 8 studies [24–31], and epinephrine plus thermal therapy in 3 studies (Fig. 2) [23, 32, 33]. All dual therapies were directly compared with epinephrine injection. Meanwhile, there was no study that directly compared dual therapies, except for one study comparing mechanical and thermal therapy. The risk of bias across studies is summarized in Additional file 2: Figure S1. As assessed by the Cochrane Risk of Bias tool, inadequate blinding provided the largest risk of bias. The qualities of the included studies were reliable overall.

### Results of the network meta-analysis

**Fig. 3:**
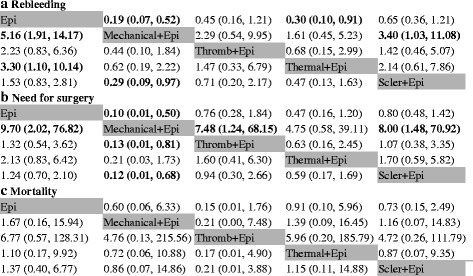
Pooled odds ratios for rebleeding, need for surgery and mortality. **a** rebleeding; **b** need for surgery; **c** mortality. The ORs were estimated in upper and lower triangles comparing the columns defined with the row-defining treatment. For study outcomes, ORs lower than 1 suggest there were beneficial comparative effects for column-defining treatments. Epi = epinephrine injection, Mech = mechanical hemostasis, Therm = thermal coagulation, Thromb = thrombin injection, Scler = sclerosant injection. Note: Significant results are in *bold*

A comparison-adjusted funnel plot for the dual therapy network showed no evidence of asymmetry regard to bleeding or surgery. The funnel plot showed some asymmetry with regard to mortality. However, Egger’s test for publication bias was not significant (*P* = 0.609). (Additional file 3: Figure S2). The contribution plot and study limitation graph were provide. (Additional file 4: Contribution plot and study limitation graph). Node-split analyses showed there was no significant inconsistency within the networks for any of the 3 outcomes (Additional file 5: Table S1). Figure 3 summarizes the results of the random-effects network meta-analysis for rebleeding, need for surgery and mortality. The combination of epinephrine plus mechanical therapy significantly reduced the rebleeding rate compared to epinephrine plus sclerosants (OR 0.29, 95% CrI 0.09–0.97) or the epinephrine injection alone (OR 0.19, 95% CrI 0.07–0.52). Epinephrine plus thermal coagulation could significantly reduce the bleeding rate compared to the epinephrine injection (OR 0.30, 95% CrI 0.10–0.91). All other comparisons showed non-significant differences in terms of rebleeding. Similarly, for the outcome of the need for surgery, epinephrine plus mechanical therapy was more effective than thrombin plus epinephrine (OR 0.13, 95% CrI 0.01–0.81), epinephrine injection (OR 0.10, 95% CrI 0.01–0.50) and epinephrine plus sclerosants (OR 0.12, 95% CrI 0.01–0.68). With respect to mortality, all comparisons among the dual therapies showed no statistical significance.

**Fig. 4:**
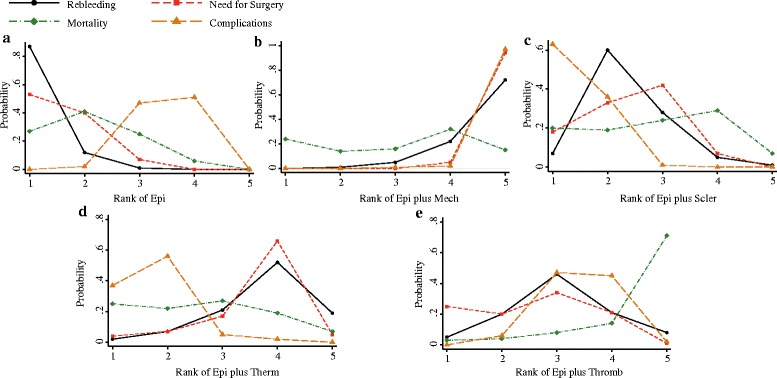
Ranking for rebleeding, need for surgery, mortality and complications of 5 interventions for bleeding ulcers. **a** epinephrine injection; **b** epinephrine injection plus mechanical therapy; **c** epinephrine injection plus sclerosant injection; **d** epinephrine injection plus thermal therapy; **e** epinephrine injection plus thrombin injection. The ranking indicated the probability to be the best treatment, the second best, the third best and so on. Rank 1 is the worst and rank N is the best

The probability of each intervention being the best treatment was ranked according to possible 5 positions (Fig. 4). Rank 5 corresponded to the highest probability of being the best treatment, and rank 1 corresponded to the worst. A ranking table was also provided (Additional file 6: Table S2). Epinephrine plus mechanical therapy and epinephrine plus thermal therapy had the highest probabilities of reducing rebleeding and the need for surgery, which suggested epinephrine plus mechanical therapy and epinephrine plus thermal therapy were more efficacious than the other remaining modalities. In addition, epinephrine plus sclerosants appeared to be associated with more complications than the other remaining treatments, whereas epinephrine plus mechanical therapy showed the best adverse effects profile.

### Results of meta-regression analyses

In meta-regression analyses, the medical treatments, the routine second-look endoscopy and year of publication were not associated with variations in logOR, in terms of rebleeding, the need for surgery, or mortality (Additional file 7: Table S3).

## Discussion

The network meta-analysis was based on 17 studies with 1939 individuals, and compared the major dual endoscopic therapies for high risk bleeding ulcers, including both benefits and complications.

Currently, hemoclips have becoming more and more widely used. Our results showed that adding mechanical therapy to epinephrine could significantly reduce the rebleeding rate and the need for surgery. As the present study was not designed to compare the efficacy of dual therapy with mechanical monotherapy, it is still not clear whether using hemoclips alone is sufficient enough for hemostasis. Previous traditional meta-analyses failed to show a superior effect of epinephrine plus hemoclips over hemoclips monotherapy. Nevertheless, there may be practical reasons to pre-inject epinephrine before other therapies for high-risk endoscopic stigmata [7, 9, 10]. Recent guidelines endorsed combination therapy as the appropriate treatment for high-risk bleeding ulcers [6, 10, 34]. Unlike thermal coagulation and sclerosants injection, the mechanical therapy also has the theoretical benefit of not inducing tissue injury [6]. Because of the lack of direct comparison evidence and the low complication rate, previous studies failed to confirm this benefit. With the consumption of ranking probability distribution, we confirmed the benefit of mechanical therapy in our study. The major restriction to the spreading of this technique would be its relatively higher cost and its requirement for a higher skilled endoscopist. Especially when applying the hemoclips to hard-to-access areas such as cardia and posterior duodenum. Future large-scale studies are needed to more clearly elucidate the risks and benefits of dual therapy for high-risk bleeding ulcers.

A traditional meta-analysis by Calvet et al. showed statistical significance in favor of using of epinephrine plus thermal coagulation for high-risk bleeding ulcers compared to epinephrine injection alone [8]. However, the analysis incorporated results from modalities that generally are not used. In this study, we did not include laser photocoagulation, which is no longer used for ulcer hemostasis because it seems to be associated with a higher risk of perforation, high cost and lack of portability [35]. Thermal coagulation is not the first choice when considering the adverse effects profile according to the rankograms. Whenever hemoclips were technically available, it would not be wrong to first consider the use of mechanical therapy, although this meta-analysis demonstrated that thermal coagulation and mechanical therapy had similar efficacy for controlling bleeding after epinephrine injection.

In previous meta-analyses, thrombin injection and sclerosant injection were indiscriminately pooled as injection therapy [7, 8, 11]. A traditional meta-analysis incorporated results from these two modalities reported that combined injection therapy decreased the rebleeding rate but did not yield a reduction in the need for surgery after initial hemostasis [7]. However, these two modalities had different characteristics. Sclerosants produce hemostasis by causing significant tissue injury and thrombosis. Injection of thrombin represents the best theoretical approach for causing thrombosis and it can create a primary tissue seal at the bleeding site [36]. In contrast, our meta-analysis assessed thrombin and sclerosants separately and progressed the field beyond conventional meta-analyses. In this meta-analysis, thrombin injection and sclerosant injection showed similar efficacy, but both of them failed to show an additional benefit for preventing rebleeding and the need for surgery. In addition, this meta-analysis was the first to evaluate the complications among five treatments. Epinephrine plus a sclerosant injection ranked the worst among the 5 treatment modalities in terms of complications. Therefore, our results demonstrated that injection of sclerosants after epinephrine injection failed to confer further benefit but may increase complication rate.

In this study, the mortality of the patients who received dual therapies did not seem to be decreased significantly. Marmo at al declared that although it was not significant, such a numerical advantage might still be important [7]. Because mortality in patients with high risk bleeding ulcers was usually associated with comorbidities, independent of the endoscopic treatment that was delivered. Nevertheless, the scarcity of the events may be another cause of non-significant results. Although the underlining mechanism was not clear, it seemed thrombin injection had the highest probability for reducing mortality. Future well-designed, large randomized controlled trials (RCTs) are needed to clarify this issue.

Our meta-analysis had several strengths. This meta-analysis compared all major dual therapies simultaneously and assessed every modality individually rather than pooling various modalities into one group. Bayesian network meta-analysis also compared therapies indirectly when there was no head-to-head trial, and obtained more precise effect estimates by assessing direct and indirect comparisons. Our study added contributions to the body of evidence indicating that different degrees of efficacy and safety exist across epinephrine-based dual therapies.

Our findings do also have some limitations. First, information about blinding was not adequately reported in the trials included in our analysis, which might have undermined the validity of the overall findings. The nature of the endoscopic treatment made blinding the participants virtually impossible. However, the rebleeding rate, need for surgery and mortality were not dependent on subjective observations [11]. Second, the year of publication of the studies included in this network meta-analysis may influence the outcomes as well as the endoscopic methods and the improved skills of the endoscopist over time. This issue always exits when conducting such a study. In addition, we did not investigate the other distribution of clinical and methodological variables in detail. For example, the percentage of the included patients who suffered from gastric ulcers and duodenal ulcers differed from study to study. This difference may provide a potential source of heterogeneity in every specific group of trials. Third, the sizes of the included studies were relatively small, although our study has established the largest sample size to individually assess the efficacy and safety of endoscopic treatments. Therefore, this network meta-analysis provides a useful and complete picture of the associations between dual therapies by using a Bayesian analytical approach.

## Conclusions

In summary, the network meta-analysis suggested that mechanical therapy was the most appropriate modality in addition to epinephrine injection. Epinephrine plus thermal coagulation was effective for controlling high-risk bleeding ulcers. There was no further benefit with sclerosants with regard to rebleeding or the need for surgery, and sclerosants were also associated with more complications than any other modality.

## Additional files

Additional file 1: Search strategy. (DOCX 37 kb)

Additional file 2: Figure S1. Cochrane risk of bias tool results. (DOCX 284 kb)

Additional file 3: Figure S2. Comparison-adjusted funnel plot for the dual therapies network. (DOCX 240 kb)

Additional file 4: Contribution plot and study limitation graph. (DOCX 258 kb)

Additional file 5: Table S1. Assessment of inconsistency between direct and indirect evidence. (DOCX 66 kb)

Additional file 6: Table S2. Ranking table. (DOCX 83 kb)

Additional file 7: Table S3. Meta-regression to explore possible confounding effects on risk of rebleeding, surgery, and mortality. (DOCX 45 kb)

## Abbreviations

ADDIS: Aggregate Data Drug Information System
CrI: Credible interval
Epi: Epinephrine injection
H_2_RA: H_2_ receptor antagonist
Hp: Helicobacter pylori
Mech: Mechanical hemostasis
NR: Not reported
OR: Odds ration
PPI: Proton pump inhibitor
PRISMA: Preferred Reporting Items for Systematic Reviews and Meta-Analyses
RCTs: Randomized controlled trials
Scler: Sclerosant injection
Therm: Thermal coagulation
Thromb: Thrombin injection
