# Supplementary material for: Systematic review with network meta-analysis: dual therapy for high-risk bleeding peptic ulcers
Source: BMC Gastroenterol. 2017 Apr 19;17:55. doi: 10.1186/s12876-017-0610-0 (PMC5395769; doi:10.1186/s12876-017-0610-0)
Supplement: Supplementary file 1 — Search strategy. (DOCX 37 kb) [file 12876_2017_610_MOESM1_ESM.docx]

**Medline (PubMed) search strategy**

#1 Gastrointestinal Hemorrhage[Mesh]

#2 Hemorrhage, Gastrointestinal[tiab]

#3 Gastrointestinal Hemorrhage*[tiab]

#4 Hematochezia*[tiab]

#5 #1 OR #2 OR #3 OR #4

#6 Hemostasis, Endoscopic[Mesh]

#7 Endoscopic Hemostas*[tiab]

#8 Hemostases, Endoscopic[tiab]

#9 #6 OR #7 OR #8

#10 #5 AND #9
