# Supplementary material for: Systematic review with network meta-analysis: dual therapy for high-risk bleeding peptic ulcers
Source: BMC Gastroenterol. 2017 Apr 19;17:55. doi: 10.1186/s12876-017-0610-0 (PMC5395769; doi:10.1186/s12876-017-0610-0)
Supplement: Supplementary file 2 — Cochrane risk of bias tool results. (DOCX 284 kb) [file 12876_2017_610_MOESM2_ESM.docx]

**Figure S1. Cochrane risk of bias tool results.** Most studies generated adequately randomized sequence. Allocations were reasonably concealed in most studies. All studies were not double blind because the endoscopist must know the type of treatment. There was no loss of follow-up in most studies, and all of them were free of reporting bias. No conflict of interest was mentioned in any of the studies.
