# Supplementary material for: Systematic review with network meta-analysis: dual therapy for high-risk bleeding peptic ulcers
Source: BMC Gastroenterol. 2017 Apr 19;17:55. doi: 10.1186/s12876-017-0610-0 (PMC5395769; doi:10.1186/s12876-017-0610-0)
Supplement: Supplementary file 3 — Comparison-adjusted funnel plot for the dual therapies network. (DOCX 240 kb) [file 12876_2017_610_MOESM3_ESM.docx]

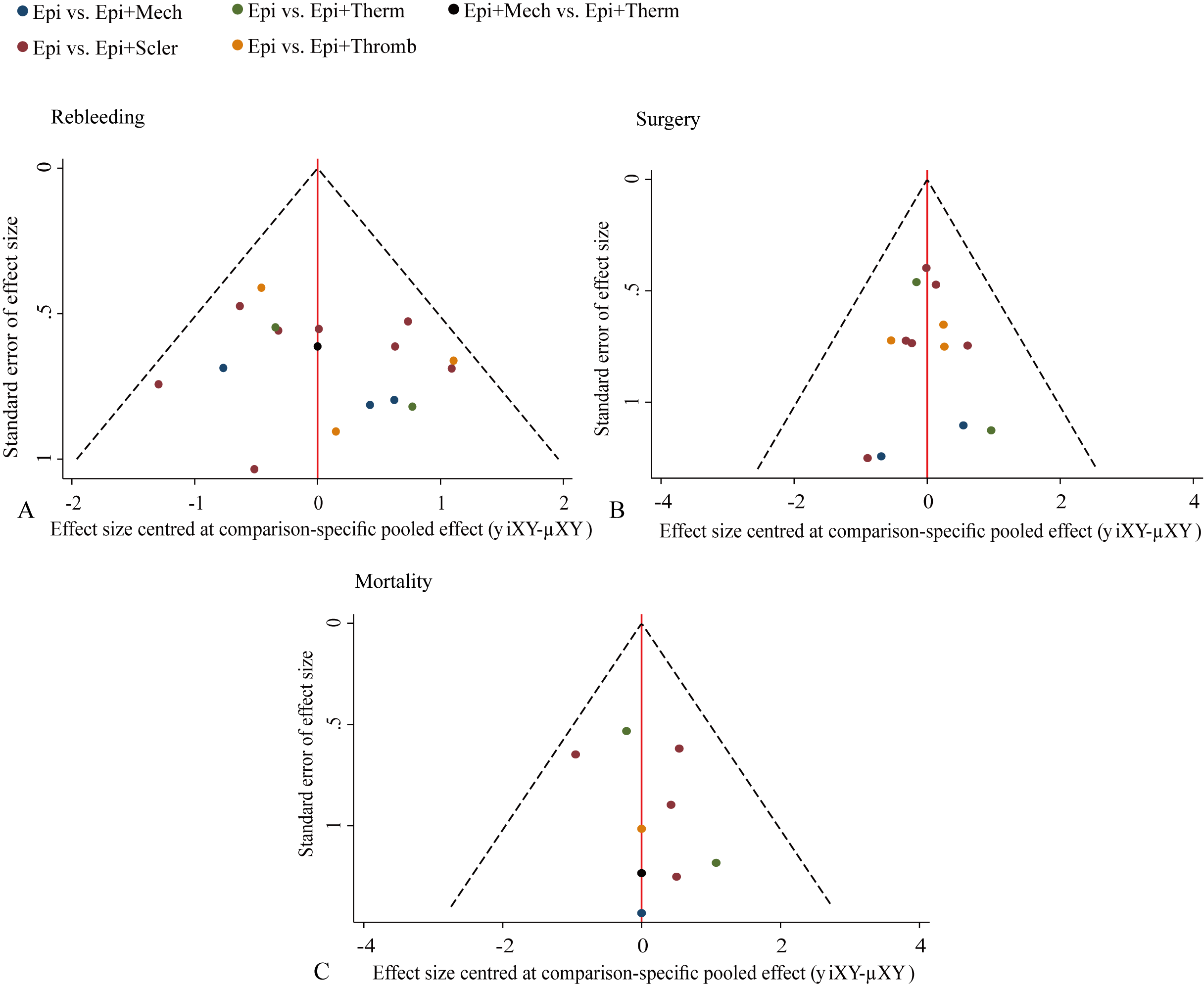
**Figure S2. Comparison-adjusted funnel plot for the dual therapies network.** (A) rebleeding, (B) need for surgery, and (C) mortality. The red line represents the null hypothesis that the study-specific effect sizes do not differ from the respective comparison-specific pooled effect estimates. Different colors correspond to different comparisons. Estimates below 1 indicate that the benefit of the experimental intervention is more pronounced in the trial than the pooled estimate. Observations from small studies missing on the right side of the line of null effect (ratio of rate ratios >1) indicate that small studies tend to exaggerate the effectiveness of experimental treatments. Epi=epinephrine injection, Mech=mechanical hemostasis, Therm=thermal coagulation, Thromb=thrombin injection, Scler=sclerosants injection.
