# Supplementary material for: Systematic review with network meta-analysis: dual therapy for high-risk bleeding peptic ulcers
Source: BMC Gastroenterol. 2017 Apr 19;17:55. doi: 10.1186/s12876-017-0610-0 (PMC5395769; doi:10.1186/s12876-017-0610-0)
Supplement: Supplementary file 4 — Contribution plot and study limitation graph. (DOCX 258 kb) [file 12876_2017_610_MOESM4_ESM.docx]

**Contribution plot and study limitation graph.**

**Figure S3. Contribution plot of the primary outcome: percentage contribution of each direct estimate to the network.** The columns represent the direct comparisons and the rows represent all possible pairwise comparisons. The sizes of the boxes are proportional to the percentage contribution of each direct summary effect for the network meta-analysis estimates (rows 1–10) and for the entire network (row 11). Epi=epinephrine injection, Mech=mechanical hemostasis, Therm=thermal coagulation, Thromb=thrombin injection, Scler=sclerosants injection.

**Figure S4. Study limitations weighted by contribution of direct estimates to the network.** The colours represent the risk of bias (green: low, yellow: moderate). Epi=epinephrine injection, Mech=mechanical hemostasis, Therm=thermal coagulation, Thromb=thrombin injection, Scler=sclerosants injection.
