# Supplementary material for: Systematic review with network meta-analysis: dual therapy for high-risk bleeding peptic ulcers
Source: BMC Gastroenterol. 2017 Apr 19;17:55. doi: 10.1186/s12876-017-0610-0 (PMC5395769; doi:10.1186/s12876-017-0610-0)
Supplement: Supplementary file 5 — Assessment of inconsistency between direct and indirect evidence. (DOCX 66 kb) [file 12876_2017_610_MOESM5_ESM.docx]

| **Table S1.** Assessment of inconsistency between direct and indirect evidence | | | | |
| --- | --- | --- | --- | --- |
| **Treatment Comparisons** | **Direct Effect** | **Indirect Effect** | **Overall** | ***P* Value of Node-Splitting Method** |
| **Rebleeding** |  |  |  |  |
| Epi, Mech+Epi | -1.38 (-2.62, -0.28) | -2.48 (-4.76, -0.24) | -1.64 (-2.65, -0.65) | 0.38 |
| Epi, Therm+Epi | -1.46 (-2.87, -0.21) | -0.42 (-2.58, 1.90) | -1.19 (-2.32, -0.10) | 0.40 |
| Mech+Epi, Therm+Epi | 1.01 (-0.84, 2.98) | -0.04 (-1.85, 1.67) | 0.48 (-0.80, 1.65) | 0.39 |
| **Need for Surgery** |  |  |  |  |
| Epi, Mech+Epi | -2.61 (-4.02, -0.80) | -14.07 (-85.03, -1.14) | -2.27 (-4.34, -0.70) | 0.14 |
| Epi, Therm+Epi | -0.86 (-2.00, 0.08) | 8.27 (-1.78, 62.31) | -0.76 (-1.86, 0.18) | 0.11 |
| Mech+Epi, Therm+Epi | 8.90 (0.81, 40.97) | 1.35 (-0.54, 3.37) | 1.56 (-0.55, 3.67) | 0.10 |
| **Mortality** |  |  |  |  |
| Epi, Mech+Epi | -0.01 (-3.09, 2.87) | -1.27 (-6.36, 3.22) | -0.51 (-2.77, 1.85) | 0.64 |
| Epi, Therm+Epi | -0.27 (-2.90, 1.89) | 0.39 (-4.11, 5.86) | -0.10 (-2.29, 1.79) | 0.80 |
| Mech+Epi, Therm+Epi | 0.70 (-2.99, 5.47) | -0.27 (-4.26, 3.30) | 0.33 (-2.39, 2.80) | 0.69 |
| Epi=epinephrine injection; Mech=mechanical hemostasis; Therm=thermal coagulation; Thromb=thrombin injection; Scler=sclerosants injection; Values were reported in a logarithmic scale. | | | | |
