# Supplementary material for: Systematic review with network meta-analysis: dual therapy for high-risk bleeding peptic ulcers
Source: BMC Gastroenterol. 2017 Apr 19;17:55. doi: 10.1186/s12876-017-0610-0 (PMC5395769; doi:10.1186/s12876-017-0610-0)
Supplement: Supplementary file 6 — Ranking table. (DOCX 83 kb) [file 12876_2017_610_MOESM6_ESM.docx]

| **Table S2**. Ranking table |  |  |  |  |  |
| --- | --- | --- | --- | --- | --- |
| **Rebleeding** | | | | | |
| **Treatment modality** | **Rank 1** | **Rank 2** | **Rank 3** | **Rank 4** | **Rank 5** |
| Epi | 0.9 | 0.09 | 0.01 | 0 | 0 |
| Mech+Epi | 0 | 0.01 | 0.04 | 0.1 | 0.85 |
| Thromb+Epi | 0.04 | 0.26 | 0.32 | 0.3 | 0.07 |
| Therm+Epi | 0.01 | 0.13 | 0.32 | 0.47 | 0.07 |
| Scler+Epi | 0.04 | 0.5 | 0.32 | 0.13 | 0.01 |
| **Need for surgery** | | | | | |
| **Treatment modality** | **Rank 1** | **Rank 2** | **Rank 3** | **Rank 4** | **Rank 5** |
| Epi | 0.54 | 0.39 | 0.07 | 0 | 0 |
| Mech+Epi | 0 | 0 | 0 | 0.02 | 0.98 |
| Thromb+Epi | 0.33 | 0.26 | 0.31 | 0.09 | 0 |
| Therm+Epi | 0.01 | 0.03 | 0.14 | 0.8 | 0.02 |
| Scler+Epi | 0.12 | 0.31 | 0.48 | 0.09 | 0 |
| **Mortality** | | | | | |
| **Treatment modality** | **Rank 1** | **Rank 2** | **Rank 3** | **Rank 4** | **Rank 5** |
| Epi | 0.24 | 0.4 | 0.29 | 0.07 | 0 |
| Mech+Epi | 0.29 | 0.14 | 0.16 | 0.32 | 0.08 |
| Thromb+Epi | 0.01 | 0.01 | 0.03 | 0.09 | 0.87 |
| Therm+Epi | 0.36 | 0.29 | 0.22 | 0.12 | 0.01 |
| Scler+Epi | 0.11 | 0.16 | 0.29 | 0.4 | 0.04 |
| **Complication** | | | | | |
| **Treatment modality** | **Rank 1** | **Rank 2** | **Rank 3** | **Rank 4** | **Rank 5** |
| Epi | 0 | 0.02 | 0.58 | 0.4 | 0.01 |
| Mech+Epi | 0 | 0 | 0.01 | 0.04 | 0.95 |
| Thromb+Epi | 0.03 | 0.05 | 0.32 | 0.56 | 0.04 |
| Therm+Epi | 0.28 | 0.64 | 0.07 | 0.01 | 0 |
| Scler+Epi | 0.68 | 0.29 | 0.03 | 0 | 0 |
| Rank 1 is worst and rank 5 is best. Epi=epinephrine injection; Mech=mechanical hemostasis; Therm=thermal coagulation; Thromb=thrombin injection; Scler=sclerosant injection. | | | | | |
