# Supplementary material for: Systematic review with network meta-analysis: dual therapy for high-risk bleeding peptic ulcers
Source: BMC Gastroenterol. 2017 Apr 19;17:55. doi: 10.1186/s12876-017-0610-0 (PMC5395769; doi:10.1186/s12876-017-0610-0)
Supplement: Supplementary file 7 — Meta-regression to explore possible confounding effects on risk of rebleeding, surgery, and mortality. (DOCX 45 kb) [file 12876_2017_610_MOESM7_ESM.docx]

| **Table S3.** Meta-regression to explore possible confounding effects on risk of rebleeding, surgery, and mortality | | | |
| --- | --- | --- | --- |
| Potential confounder | Rebleeding | Surgery | Mortality |
|  | *P* value | *P* value | *P* value |
| Medical treatments | 0.663 | 0.242 | 0.901 |
| Whether use routine second-look endoscopy or not | 0.087 | 0.778 | 0.876 |
| Year of publication | 0.872 | 0.218 | 0.266 |

Medical treatments include PPI and H_2_RA. PPI=Proton pump inhibitor; H_2_RA=H_2_ receptor antagonist.
